# A Closer Look at the “Right” Format for Clinical Decision Support: Methods for Evaluating a Storyboard BestPractice Advisory

**DOI:** 10.3390/jpm10040142

**Published:** 2020-09-23

**Authors:** Brian J. Douthit, R. Clayton Musser, Kay S. Lytle, Rachel L. Richesson

**Affiliations:** 1 School of Nursing, Duke University, Durham, NC 27710, USA; rachel.richesson@duke.edu; 2 School of Medicine, Duke University, Durham, NC 27710, USA; clay.musser@duke.edu; 3 Duke Health, Duke School of Medicine, Durham, NC 27710 USA; kay.lytle@duke.edu; 4 Department of Biostatistics and Bioinformatics, Duke University, Durham, NC 27710, USA

**Keywords:** clinical decision support, systems evaluation, alert fatigue

## Abstract

(1) Background: The five rights of clinical decision support (CDS) are a well-known framework for planning the nuances of CDS, but recent advancements have given us more options to modify the format of the alert. One-size-fits-all assessments fail to capture the nuance of different BestPractice Advisory (BPA) formats. To demonstrate a tailored evaluation methodology, we assessed a BPA after implementation of Storyboard for changes in alert fatigue, behavior influence, and task completion; (2) Methods: Data from 19 weeks before and after implementation were used to evaluate differences in each domain. Individual clinics were evaluated for task completion and compared for changes pre- and post-redesign; (3) Results: The change in format was correlated with an increase in alert fatigue, a decrease in erroneous free text answers, and worsened task completion at a system level. At a local level, however, 14% of clinics had improved task completion; (4) Conclusions: While the change in BPA format was correlated with decreased performance, the changes may have been driven primarily by the COVID-19 pandemic. The framework and metrics proposed can be used in future studies to assess the impact of new CDS formats. Although the changes in this study seemed undesirable in aggregate, some positive changes were observed at the level of individual clinics. Personalized implementations of CDS tools based on local need should be considered.

## 1. Introduction

Since the inception of electronic health records (EHRs), clinical decision support (CDS) has been a vital tool in supporting information delivery and has acted as a powerful resource to clinicians at the point-of-care. CDS has evolved beyond its humble beginnings; once primarily used for simple tasks such as allergy checking, more recent applications have used advanced computational methodologies, such as machine learning and deep learning to support clinical processes [1,2,3,4]. Today, new applications of CDS are being used to advance personalized medicine with the discovery of new knowledge in “-omic” research [5]. Such advances in personalized treatments need CDS as a platform for delivery, necessitating the continual improvement and evaluation of CDS systems to ensure success.

Guidance is available to assist in CDS evaluation, but the “five rights” of CDS have become a common set of principles among informaticians. First articulated by Osheroff et al., in 2007 [6], and now recognized by organizations such as the Agency for Healthcare Research and Quality (AHRQ), the five rights state that successful CDS tools communicate the right information, to the right person, in the right format, through the right channel, at the right time in the workflow:

1. Right Information: CDS should be relevant and supported by the best evidence available.
2. Right Person: CDS should be targeted to the correct individuals and support their workflow.
3. Right Format: CDS should be presented in a way that is useful to the task at hand, potentially as alerts, order sets, or info buttons, depending on the application.
4. Right Channel: CDS should be delivered through a medium that is most appropriate for its goal, whether through the EHR, paper flowsheets, or mobile devices.
5. Right Time in the Workflow: CDS should be available to the user when it is needed.

While the five rights are comprehensive, they are meant to be used as general guidance and not as prescriptive instruction. There is opportunity for more guidance on how to address each of the five rights, but in particular, format is important, because it may interfere with the other four rights if not optimized. For example, while it is simple in most cases to decide between an order set (a templated set of orders that may be needed for a specific clinical scenario) and an alert, the type of alert needed for a given situation is not as clear. Alerts usually range from “passive” alerts (those that appear but do not physically interrupt workflow) to more “active” alerts (pop-up alerts that generally do not let the user continue until they are addressed). However, there is a lack of formal delineation between different types and their nuances, further complicating CDS evaluation methods. The lack of study in this area can be explained by the fact that EHRs vary by vendor and, historically, have been largely customized to each health system. Differences existed not only between established vendors and homegrown models, but also between implementations from a single vendor. While this holds true in many cases today, strategies to narrow the gap between EHR implementations are in place [7]. For instance, the Epic© (Madison, WI, USA) EHR uses alerts known as BestPractice Advisories (BPAs) with similar features across all sites. In this case, the types of triggers than can be used by alerts are standard. The literature, however, does not give specific guidance on how to best choose triggers and other features of a BPA based on its goal.

As an example, an institution would not want to implement all BPAs as pop-up alerts, as this could cause extreme alert fatigue and devalue alerts critical to patient safety. Instead, many BPAs are designed to be “passive”, displayed in a section of the EHR to be viewed by the user without interruption. The format of the alert has major implications for the effectiveness of the BPA, and should be carefully considered and tested. However, the decision to implement an interruptive or non-interruptive BPA is not a binary choice; hybrid options and new formats have become available. The addition of these new formats further complicates the choice of CDS alert format and the selection of methods for evaluation.

Current methods for evaluating CDS are limited by the non-standardized ways we define their behavior, describe their success, and report their outcomes. A major challenge is the lack of standard terminologies and operationalized definitions relating to CDS format, especially in how we describe the “interruptiveness” or “urgency” of the format. A new method of BPA delivery in Epic© EHRs has recently become available via the “Storyboard” component of the chart. Where some BPA formats are truly passive and some are interruptive pop-up alerts, BPAs that appear in the Storyboard would not be deemed interruptive (since they do not pop up) but do have the potential to be more eye-catching and persistent than traditional passive alerts, displaying a colored text banner that remains visible as the user navigates the patient’s entire chart. By alerting the user to the need to address a BPA, a moderate level of urgency may be communicated to the user without interrupting workflow.

Duke Health recently began using the Storyboard to trigger a select number of important BPAs. One such BPA was designed to encourage an assessment of patients’ learning preferences (referred to as the “learning screen”). This BPA is an example of a tool that should improve the collection of personalized data, that could then be used to tailor treatments and interventions. However, this BPA has traditionally been difficult to manage. From its inception, user response to the BPA has been less than ideal, as evidenced by persistent incomplete assessments, despite very high firing rates (dwarfing other BPAs, in fact) and concern for alert fatigue. First implemented in an interruptive, pop-up format and subsequently in a passive format (but still required via pop-up to be completed before closing the chart) without satisfactory results, this BPA appeared to be a prime candidate to be tested with the Storyboard format.

To begin developing methodologies for assessing Storyboard BPAs, we conducted a retrospective cohort study to assess the Storyboard format’s influence on the learning screen BPA. Unfortunately, due to the timing and effects of the COVID-19 pandemic, the results of our analysis cannot be used to make conclusions about the effectiveness of the BPA or Storyboard in general. Rather, this study serves as an archetype methodology to assess the influence of format change. Therefore, the primary goal of this study is to demonstrate and discuss an approach to evaluating the effect of format changes on BPAs.

## 2. Materials and Methods

### 2.1. Framework and Outcomes

**Figure 1:**
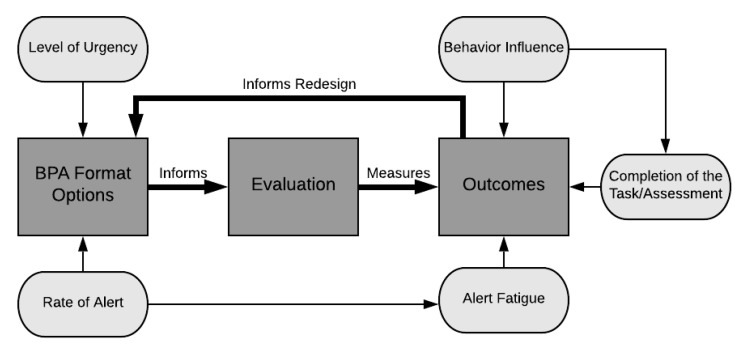
Alert Format Evaluation Model.

To begin, the concept of BPA “effectiveness” must be defined and operationalized so that we may have a clear path of how to analyze the impact of the changes that were made to the format of the learning screen BPA. Concepts from the framework for evaluating the appropriateness of clinical decision support alerts and responses by McCoy et al. [8] (level of alert urgency) and the learning health system model [9] (feedback loop between evidence and design) were combined with the structure-process-outcome format of the Donabedian Model [10], resulting in an “Alert Format Evaluation Model” (Figure 1).

The goal of our evaluation was to test whether an increased level of urgency (the addition of a persistent, yellow banner in the Storyboard) and an increased number of BPA firings contributed to better outcomes. To do this, we defined three outcome domains: alert fatigue, behavior influence, and task completion. Alert fatigue is crucial to assess with the implementation of all CDS tools, as it has been associated with medical errors and negative patient outcomes [11]. Behavior influence refers to how the user interacts with the BPA. While task completion (in our case, the completion of the learning screen, our clinical outcome) is the ultimate goal, understanding how the user interacts with the BPA may give insights into data quality. In other words, even if changing the BPA format does not affect task completion, an increase in meaningful responses (i.e., improving data quality) may be just as important. These three domains together can help to better explain the effectiveness of the BPA format changes on learning screening, by not only assessing completion rates, but also weighing the other components in relation to any perceived gain.

### 2.2. Learning Screen BPA Behavior and Redesign

**Figure 2:**
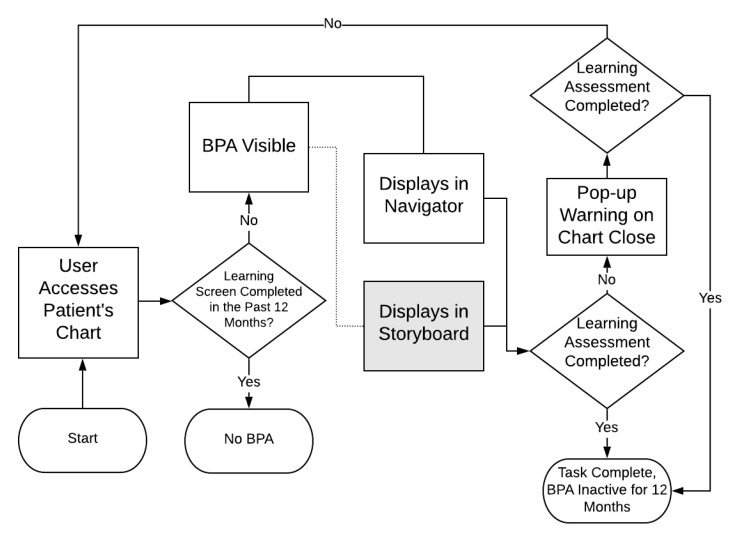
Learning Screen BestPractice Advisory (BPA) Functionality.

The BPA selected for this study is the learning screen, which triggers in an outpatient encounter when the patient has not had a learning screen in the past 12 months, and therefore has raised concerns about alert fatigue due to a high volume of firings in recent years. The BPA directs nursing staff (via a hyperlink) to an education activity with five questions that assess learning preferences, addressed to either the patient or their caregiver. In the pre-redesign state, this BPA was found only in the “navigator” screen, an area voluntarily accessed by the user to complete passive BPAs. However, if not completed by the time the user closes the chart, the BPA will fire as a pop-up reminder. The redesign of this BPA using Storyboard included the same functionality, but with the addition of a persistent yellow banner at the upper left of the chart. Figure 2 represents the functionality of the BPA pre- and post-redesign; the grey box indicates the Storyboard format that was added.

### 2.3. Data Sources and Measures

To measure the three domains of alert fatigue, behavior influence, and task completion, we determined which data were available that could contribute to their assessment. Our primary data sources include the BPA cube, which is an Epic© data structure that allows for querying many aspects of the firing of specific BPAs (including buttons clicked, free text responses, unique patients, etc.), and an existing operational report that tabulates the completion of the learning screen by clinic. Table 1 summarizes the measures, their definitions, and their sources, as categorized by the associated domain.

To assess alert fatigue, the number of firings and the alert to patient ratio were used as surrogate measures. The raw number of firings is not particularly effective on its own to explain alert fatigue, as non-interruptive alerts can often appear multiple times as the user’s navigation of the chart causes the BPA to refresh. It would also be influenced by the fluctuations in the numbers of patients being seen due to the COVID-19 pandemic. However, this metric is included, as it is often a metric used by organizations to identify troublesome BPAs. Alert to patient ratio should approximate alert fatigue more closely, especially for storyboard BPAs. Here, we gain a more temporal view into the exposure of the yellow banner, as when there are a greater number of firings per patient, there is a probable increase in the amount of exposure to the alert.

Behavior influence, or how users interact with the BPA, is measured by activity link selection ratio and erroneous free-text ratio. Clicking the activity hyperlink is the preferred outcome of the alert, since this suggests that the user took the suggestion and chose to perform a learning screen. Therefore, understanding the average ratio of activity link clicks per patient gives insight into the BPAs influence to open the relevant flowsheet. Erroneous free text refers to override responses that appear to be workarounds, for example, due to an interruption in workflow after which the user types “.”, “0”, “L”, or “123”. We hypothesized that, if providers interact with the BPA at the right time in the workflow, then the number of these responses will decrease.

Task completion is a measure of the actual completion of the learning screen assessment (rather than just clicking the activity link) and the quality of its completion. Ratio of task completion is a measure of how many assessments were completed compared to the total number of assessments that needed to be completed. To further this assessment, the task completion quality ratio used the final question of the assessment as a quality proxy, giving a ratio between the number of assessments marked as “completed” and the number of assessments that answered the fifth and final question. This report was already available at the time of this study, having been developed due to documentation differences between inpatient and outpatient areas. The ability to check for the completion of all five questions was not available at this time.

### 2.4. Setting and Population

This study used data collected from users working in Duke Health outpatient clinics. Duke Health is a large academic medical center based in Durham, North Carolina, with many outpatient clinics across the state. Overall, 686 clinics were included in the analysis, as these clinics had data for the pre- and post-redesign phases. Users able to complete these assessments include medical assistants, registered nurses, licensed practical nurses, and medical technicians. No formal training was given regarding the learning screen BPA; rather, tip sheets and announcements on the Epic Learning Home dashboard provided information about Storyboard in general.

### 2.5. Study Design

This study used a retrospective cohort design to assess BPA format effectiveness. The study date range, from 09 October 2019 to 30 June 2020, resulted in 133 days (19 weeks) of data before, and 133 days after, the Storyboard go-live date of 19 February 2020. Shortly after go-live, COVID-19 began to significantly impact American healthcare systems. Due to this timing, we cannot make causal inferences from the data with confidence. Therefore, readers of this manuscript should focus primarily on the methods for assessment; results are shared with strong caveats about interpretation and to exemplify how data can be gathered and analyzed.

Alert fatigue and behavior modification were assessed at a system level (aggregate data of all clinics), while task completion was assessed at both a system and clinic level. Clinic level data for alert fatigue and behavior influence measurements were not available. Data for each measurement were taken from the BPA cube and the Epic© report for learning task completion. To assess for the effectiveness of the BPA format change, we ultimately expect to see significant differences in the task completion rates. The other domains were considered in the context of the level of change, adding to the discussion and process of this evaluation. This study used non-identifiable data and was determined exempt by the Duke IRB.

### 2.6. Analysis

Calculations were completed as noted in Table 1 for both pre- and post-redesign. As previously noted, task completion was assessed at both a system and clinic level, with calculations outlined in Table 1. However, we report an additional metric to assist in the interpretation of task completion. We categorized task completion percentage into four categories: Low (0–4%), Medium-Low (5–49%), Medium-High (50–94%), and High (95–100%). As an organizational goal, we expect some level of non-completion, even in an ideal environment, due to emergencies and other issues. Therefore, a 95–100% completion rate is considered satisfactory. With this insight and influence from Rogers’ theory of diffusion of innovations [12], we created the remaining categories to reflect levels of BPA completeness. This included a small group of those who are “laggards” (essentially non-compliant, according to Rogers), with the remaining users split equally into the “early majority” (medium–high) and the “late majority” (medium–low). At the level of individual clinics, this should be helpful to track meaningful changes towards organizational goals for this BPA.

Descriptive statistics are reported below. Paired *t*-tests or Wilcoxon rank-sum tests were done to assess the differences between pre- and post-implementation. Post-implementation scores are also displayed to show proportions between the pre- redesign and Storyboard formats. Clinic completion categories were calculated, and any improvements post-implementation were summarized. SAS version 9.4 (Cary, NC, USA) was used for the analysis.

## 3. Results

### 3.1. Alert Fatigue

During the study period, the BPA fired a total of 1,107,981 times, with 257,446 occurring in the first 19 weeks (pre-intervention) on 211,756 patients, and 850,535 occurring in the second 19 weeks (post-intervention) on 173,346 patients. Skewness for differences in fires between the pre- and post-study period was 0.02, allowing for a paired *t*-test to be conducted to test for differences in the fire rates and fire to patient ratios. Statistics for both number of fires and alert to patient ratio are summarized in Table 2. Both number of firings and alert to patient ratio were significantly higher post-implementation, with weekly firings increasing from 12,872 to 42,527 (*p* < 0.0001), and the alert to patient ratio increasing from 1.12 to 4.92 firings per patient (*p* < 0.0001).

### 3.2. Behavior Influence

During the study period, the activity link was clicked 258,910 times. Both pre- and post-, the average click per patient remained the same, at 0.67. No statistical difference was found between these ratios (*p* = 0.97). Of the 361,852 text responses, 2308 were considered erroneous, with 1322 (0.6%) pre-implementation and 986 (0.5%) post-implementation. This 20% relative decrease from the pre- to post-redesign phase was statistically significant (*p* = 0.0431), but it may not represent operational significance as the percentages are so low. Statistics for both activity link selection ratio and erroneous free-text ratio are summarized in Table 3.

### 3.3. Task Completion

To calculate task completion, we assessed both overall completion and quality rates. In addition, we also noted the number of clinics that had significant improvement post-implementation. In total, 686 clinics were assessed. Clinics were excluded if they did not have data during both the pre- and post-implementation periods. Wilcoxon rank sum test was used because distributions of the pre- and post-differences for the two metrics were noted to be skewed. In both task completion and quality, there was a significant decrease after the implementation of Storyboard (Table 4); the average task completion decreased from 58% to 40% (*p* < 0.0001), and completion quality (as measured by completing the final screening question) decreased from 41% to 27% (*p* < 0.0001).

To further assess task completion, data on individual clinics were analyzed. Of 686 clinics, 104 were already achieving a high (95–100%) rate of assessment completion pre-implementation, but this number dropped to 47 clinics post-implementation. Table 5 further summarizes categories pre- and post-implementation. Of note, post-implementation, 98 clinics (14%) had higher rates of completion (with an average completion increase of 13%), and 29 (4%) of those had moved to a higher category (Table 5).

## 4. Discussion

### 4.1. Assessment of Format Effectiveness

Even with the confounding factors related to COVID-19, alert fatigue may have increased with the addition of Storyboard, both in terms of number of alerts and alerts per patient. However, these results are not surprising, as the intent was to increase visibility with the Storyboard format; we suspect a large proportion of the firings are due to user navigation events which cause the BPA to refresh and re-fire. This informs the results of the other two domains, allowing us to ask the question: Is the potential increase in alert fatigue worth the outcome? In this case, it likely is not, but this question would be worth asking even if the results had been positive. The risk of alert fatigue here is likely much less than a more typical change of an alert from a passive notification to active pop up, but a lack of formalized definitions for levels of alerting makes evaluating alert fatigue based on format change difficult. Additionally, although our data cannot be used to make strong inferences, it does appear to be consistent with the findings of other studies that an increase in alert fatigue can have negative impacts on outcomes [13]. Careful consideration must be taken when deciding to increase the urgency of CDS tools, as even if the effects are not apparent early, there is a possibility of worsening outcomes over time. Assessments such as this one should be completed on a regular basis, even after the initial implementation period has ended.

Behavior influence was assessed due to its strong ties to both alert fatigue and the outcome of task completion. As the level of urgency increases, we would expect more interaction with the CDS tool. In this case, there was no apparent difference pre- and post-implementation, and the amount of interaction with the Storyboard was minor in comparison to accessing the BPA through the older format. This does make sense, even in light of the historical effects of COVID-19, as the BPA was required on chart close in both pre- and post-format. There was a modest decrease in erroneous free-text responses, potentially indicating that those who interacted with the storyboard BPA did so at a time that did not interrupt their workflow. Regardless, this result further shows the importance of format on the intersection of workflow timing and data quality. Although our results indicate that this implementation of a Storyboard BPA was not successful overall, there may be other cases where the numeric outcome does not significantly improve, but the quality of the responses do. This may be important in some CDS tools, especially when the data collected will be used for research or process improvement where data quality is essential. This is also important to consider in personalized health, as such interventions require data that are sensitive to the variance of the individual; if the data quality is not adequate, this may result in an ineffective intervention or, worse, patient harm.

Finally, our assessment of task completion showed that overall, adding this BPA to the Storyboard appeared not to have the intended results. At an organizational level, this may seem a failure due to the apparent significant decrease in completion and quality. However, we also noted that 14% of the clinics improved their completion rates, with 4% improving in their completion category. To dismiss these effects may be imprudent, as even a modest improvement in a single site performance may be worth the format change, especially if the BPA is critical to patient safety. It is also important to note that task completion itself is still a proxy to desired outcomes; we would hope that documenting patients’ learning preferences and abilities would lead to better education-related outcomes, but these are difficult to measure. Health systems should use caution when using such data as a proxy, but such evaluations are still valuable when more direct measures of health outcomes are not available.

### 4.2. Personalizing BPA Implementations

The 13% improvement in completion seen by those clinics who improved may be clinically substantial, but it is at risk of being overlooked in the context of the decreased performance in aggregate. In future CDS implementations, we recommend that health systems consider a more personalized selection of tools based on individual or clinic-level need. With modern data tools, it appears feasible to assess clinic-level improvements and decide whether to keep or retire a BPA based on their performance. While adding and removing individual clinics would likely increase the technical complexity of the BPA behind the scenes, an improvement in both task completion and alert fatigue may make the extra effort worthwhile. One-size-fits-all metrics of BPA success may fail to capture the nuances of impact at local levels within a large healthcare organization, and future work should address factors that could lead to an intervention being helpful in one locality and detrimental in another. In the future, and in the spirit of personalized medicine, such assessments should become even more granular—even at the level of the individual clinician—to support care and deliver the best outcomes possible.

### 4.3. Limitations

This study used data collected from 9 October 2019 to 30 June 2020, providing an equal amount of time pre- and post-implementation. Unfortunately, the COVID-19 pandemic has significantly affected health systems across the world and may have introduced significant historical effects during the post-redesign phase. Effects could include significant changes in volume of patient visits, in the availability of nursing staff to attend to functions like learning screening, and others. Starting in March 2020, Duke Health saw a large decrease in ambulatory visits before converting rapidly to telehealth visits, via telephone or video. There was dramatic growth of telehealth compared to pre-COVID usage. Using telehealth, Duke Health saw patients across North Carolina, all 50 states, Washington DC, and two US territories. The patient characteristics of race, gender, age, and payer mix for telehealth visits remained consistent with those of in-person visits.

This Storyboard format of BPA is a new feature in the Epic EHR, and an example of its use and evaluation could be valuable to other organizations. The passive education for Storyboard and lack of specific education about this Storyboard BPA may have influenced the results. User-level data could not be feasibly accessed for use in our analysis. Future work that uses randomized testing of format changes with qualitative components could help us better understand the user experience and target areas for improvement. Number of total users and alerts-per-user ratios could have strengthened the analysis. In addition, the implementation did not reflect true A-B testing; rather, the BPA was added to Storyboard, resulting in all users being exposed to both formats. To evaluate format change, randomized A-B testing would be ideal, as the remaining functionality of the previous implementation likely reduced our ability to detect true influences of the format change. We hope that this methodology for assessing a single BPA’s format change is useful as a guide for future similar evaluations. It is also important to note that the Storyboard itself is a new chart-viewing option that provides a vast array of features beyond the display of BPAs, and, anecdotally, it has been overwhelmingly well received at our institution.

## 5. Conclusions

Assessing CDS tools is a complicated, multi-faceted task. Even in isolation, a single CDS tool requires much effort to fully understand its effects, and when assessing multiple factors (often hundreds in larger hospital systems), this task becomes even more difficult. Planning for prospective evaluation of a CDS tool should be included as part of its development, with metrics relevant to its success identified. As seen in this example, format evaluation can help to understand the impact of the tool on organizational goals. Furthermore, it is important to move toward assessments that are more personalized, at the level of individual clinics or even individual clinicians. Using a one-size-fits-all assessment of CDS can overlook a tool’s positive effects in some contexts. In other words, there may be benefits of using a tool in some areas and not others. Over time, these small improvements may add up to improved quality of care and other organizational outcomes. In summary, CDS evaluation should begin to reflect the values of personalized medicine, taking individualized approaches to assessment and implementation to maximize CDS effectiveness at local levels, even when aggregate metric goals are not achieved.

## Figures and Tables

**Table 1:** Measures Used to Evaluate the Effectiveness of the Storyboard Format on the Learning Screen BPA.

	Measure Name	Definition	Calculation
**Alert Fatigue**	**Number of Firings**	Number of times the BPA appears	Integer
	**Alert to Patient Ratio**	Average number of firings per patient	# of firings ÷ # of unique patients
**Behavior Influence**	**Activity Link Selection Ratio**	Number of times the activity link (embedded hyperlink to the assessment) is selected per patient	# of activity link clicks ÷ # of unique patients
	**Erroneous Free-Text Ratio**	Ratio of free-text responses that are illogical (e.g., numbers, random letters)	# of erroneous free-text responses ÷ all free-text responses
**Task Completion**	**Ratio of Task Completion**	Number of assessments completed out of the total possible	Assessments ÷ total possible assessments
	**Task Completion Quality Ratio**	Number of assessments completed with the final question answered	Assessments with final question answered ÷ total possible assessments

**Table 2:** Alert Fatigue Pre- and Post-Storyboard Implementation.

Measure	Study Period	N	Mean	Std Dev	Min	Max
**Number of Firings**	Pre	257,446 firings	12,872 firings/week	4660	3252	18,884
	Post	850,535 firings	42,527 firings/week	19,155	17,266	69,526
	Total	1,107,981 firings	*p* < 0.0001 α = 0.05			
**Alert to Patient Ratio**	Pre	257,446 patients	1.21 firings/patient	0.03	1.15	1.26
	Post	173,346 patients	4.92 firings/patient	0.46	3.14	5.23
	Total	385,102 patients	*p* < 0.0001 α = 0.05			

**Table 3:** Behavior Influence Pre- and Post-Storyboard Implementation.

Measure		N	Mean	Std Dev	Min	Max
**Activity Link Selection Ratio**	Pre	142,005 clicks	0.67 clicks/week	0.02	0.63	0.70
	Post	116,905 clicks (112,096 old format, 4809 new Storyboard)	0.67 clicks/week	0.02	0.60	0.70
	Total	258,910 clicks (254,101 old format, 4809 new Storyboard)	*p* = 0.97 α = 0.05			
**Erroneous Free-Text Ratio**	Pre	1322 erroneous responses	0.006 (0.6%)	0.002	0.004	0.010
	Post	986 erroneous responses	0.005 (0.5%)	0.001	0.004	0.010
	Total	2308 erroneous responses	*p* = 0.0431 α = 0.05			

**Table 4:** Task Completion Pre- and Post-Storyboard Implementation.

Measure		N (Assessments)	Mean	Std Dev	Min	Max
**Ratio of Task Completion**	Pre	129,319 completed/222,964 required	0.58 (58%)	0.36	0	1
	Post	63,644 completed/159,111 required	0.40 (40%)	0.32	0	1
	Total	511,394 completed/382,075 required	*p* < 0.0001 α = 0.05			
**Task Completion Quality Ratio**	Pre	91,415 fully completed/222,964 total	0.41 (41%)	0.33	0	1
	Post	42,960 fully completed/159,111 total	0.27 (27%)	0.28	0	1
	Total	134,375 fully completed/382,075 total	*p* < 0.0001 α = 0.05			

**Table 5:** Task Completion by Clinic.

**Completion Category**					
**Study Period**	**Low** **(0–4%)**	**Medium-Low** **(5–49%)**	**Medium-High** **(50–94%)**		**High** **(95–100%)**
**Pre**	110 clinics	159 clinics	313 clinics		104 clinics
**Post**	147 clinics	275 clinics	217 clinics		47 clinics
**Change Metrics**					
**Score Change**		**# of Clinics that Improved in Score**		**# of Clinics that Decreased in Score**	
		98 of 686 clinics (14%)		505 of 686 clinics (74%)	
**Average Increase in Completion in Clinics that Improved Post-Redesign**		13% improvement			
**Category Change**		**# of Clinics that Improved in Category**		**# of Clinics that Decreased in Category**	
		29 of 686 clinics (4%)		277 of 686 clinics (40%)	
